# Understanding the Adverse Hemodynamic Effects of Serious Thoracic Injuries During Cardiopulmonary Resuscitation: A Review and Approach Based on the Campbell Diagram

**DOI:** 10.3389/fphys.2019.01475

**Published:** 2019-12-03

**Authors:** Youcef Azeli, Juan Víctor Lorente Olazabal, Manuel Ignacio Monge García, Alfredo Bardají

**Affiliations:** ^1^Sistema d’Emergències Mèdiques de Catalunya, Barcelona, Spain; ^2^Emergency Department, Sant Joan University Hospital, Reus, Spain; ^3^Institut d’Investigació Sanitari Pere Virgili, Tarragona, Spain; ^4^Clinical Management Anaesthesiology Unit, Resuscitation and Pain Therapy, Juan Ramón Jiménez Hospital, Huelva, Spain; ^5^School of Medicine and Health Sciences, International University of Catalonia (IUC), Barcelona, Spain; ^6^Intensive Care Department, University Hospital of SAS Jerez, Jerez de la Frontera, Spain; ^7^Department of Cardiology, Joan XXIII University Hospital, Tarragona, Spain; ^8^School of Medicine, Rovira i Virgili University, Tarragona, Spain

**Keywords:** cardiopulmonary resuscitation, adverse effect, thoracic injuries, hemodynamics, Campbell diagram

## Abstract

Chest compressions during cardiopulmonary resuscitation (CPR) generate cardiac output during cardiac arrest. Their quality performance is key to achieving the return of spontaneous circulation. Serious thoracic injuries (STIs) are common during CPR, and they can change the shape and mechanics of the thorax. Little is known about their hemodynamic effects, so a review of this emerging concept is necessary. The Campbell diagram (CD) is a theoretical framework that integrates the lung and chest wall pressure-volume curves, allowing us to assess the consequences of STIs on respiratory mechanics and hemodynamics. STIs produce a decrease in the compliance of the chest wall and lung. The representation of STIs on the CD shows a decrease in the intrathoracic negative pressure and a functional residual capacity decrease during the thoracic decompression, leading to a venous return impairment. The thorax with STIs is more vulnerable to the adverse hemodynamic effects of leaning, hyperventilation, and left ventricular outflow tract obstruction during CPR. A better understanding of the effects of STIs during CPR, and the study of avoidable injuries, can help to improve the effectiveness of chest compressions and the survival in cardiac arrest.

## Introduction

The survival of patients who suffer cardiac arrest is barely over 10%, and improving this represents a great challenge (Gräsner et al., 2016). Thoracic compressions ensure cardiac output during cardiopulmonary resuscitation (CPR). That is why they are the most important maneuver performed during CPR and should be performed as early as possible without fear of causing harm to the patient. Chest compressions during CPR should be started by placing the heel of the hand over the lower half of the sternum. The guidelines recommend compressions at a depth between 5 and 6 cm for a medium-sized adult, at a frequency of 100 per min, and ensuring the return of the sternum to its original position in the decompression phase (Perkins et al., 2015).

Thoracic injuries secondary to CPR are common (Kralj et al., 2015). The depth of chest compressions, advanced age, female sex, and longer CPR duration are the main risk factors for a thoracic injury (Ram et al., 2018), and they have been associated with a low rate of return of spontaneous circulation (Kashiwagi et al., 2015). The use of mechanical compressors has become widespread, but this has not led to improved survival from cardiac arrest (Gates et al., 2015), and several studies have described an increased incidence of serious thoracic injuries (STIs) after their use (Smekal et al., 2014; Koster et al., 2017). Otherwise, CPR assisted by mechanical compressors allows prolonging the resuscitation and maintaining its quality. This would allow the initiation of advanced therapies when indicated—such as CPR with extracorporeal membrane oxygenation (ECMO) (Roncon-Albuquerque et al., 2018).

The generation of cardiac output during CPR is mainly based on two complementary theories: the theory of the thoracic pump and the theory of the cardiac pump (Chalkias et al., 2019). The sternal recoil during the decompression phase decreases intrathoracic pressure, and this is crucial to maintaining venous return and therefore cardiac output. During CPR, a thoracic molding and a worsening of thoracic biomechanics secondary to thoracic injuries can be produced (Beesems et al., 2015; Oh, 2017). However, the adverse hemodynamic effects of this phenomenon are an emerging concept scarcely studied. A Campbell diagram (CD) is a theoretical model that integrates pressures, the chest wall and lung volumes and allows us to understand the physiology and mechanics of the respiratory system as a whole and its hemodynamic effects under several pathophysiological conditions (Vassilakopoulos, 2008). This state-of-the-art review summarizes the evidence available in the literature about the effects of STIs on thoracic mechanics and uses a theoretical CD-based approach to understand the associated hemodynamic effects.

### Thoracic Molding, Injuries, and Biomechanics During Cardiopulmonary Resuscitation

Repeated compressions during CPR produce thoracic molding. These changes in the thoracic cage range from the dislocation or subluxation of the chondrosternal joints to multiple and bilateral rib fractures (Kralj et al., 2015). The effects may range from a decreasing stiffness of the sternal hinge to a significant reduction in the anteroposterior diameter due to a flattening or collapse of the thoracic wall (Liao et al., 2010).

Rib fractures related to CPR are commonly located along the anterior axillary line, and the most affected ribs are the third to the sixth. Two-thirds of a series of non-survivors of an attempt at manual CPR presented serious rib cage damage; among them, 87% presented bilateral rib fractures (Azeli et al., 2019). In a randomized clinical trial designed to assess the safety of mechanical chest compressions, the incidence of STIs ranged from 39.8 to 45.6%, with eight being the mean number of fractured ribs in the mechanical chest compression groups. In a prospective autopsy cohort, 41.2% of the patients presented an intrathoracic injury strongly correlated with skeletal chest fractures, with lung and heart contusions being the most common (Ihnát Rudinská et al., 2016).

The force used during chest compression is transmitted to the sternal hinge, which helps to create intrathoracic pressure variations during the compression-decompression phase, causing an extrathoracic anterograde flow. The sternal hinge is strongly fixated at its cephalic end by the sternoclavicular joints. In contrast, the cartilages of the caudal sternocostal joints are more flexible and assist the sternum’s ventral movement (Pickard et al., 2006). During thoracic compression, the cartilage-rib system, on one hand, and the intrathoracic viscera, on the other, reduce the impact of the force received and help to restore the sternum back into its original position.

The physical proprieties of the rib cage are elastoplastic and can be depicted in a force vs. displacement curve that has two parts (Schafman et al., 2016). The first part of this curve represents its elastic component and is linear until the elastic limit is reached. This part allows to calculate chest stiffness as the ratio between force and displacement. When the applied force exceeds the elastic limit, the plastic component of the curve starts. From this point, the material will not recover its original shape and size and it is susceptible to breakage.

In a series of 91 out-of-hospital cardiac arrest patients, Tomlinson et al. demonstrated that the chest stiffness, measured as the average force needed to reach 25 mm of sternal compression depth, decreased with the number of compressions (Tomlinson et al., 2007). These authors suggested that this decreased stiffness could be due to the loss of the integrity of the elastic properties of the chest secondary to fractures of the ribcage. An example of this is the sharp drop of the chest compression release velocity measured by an accelerometer in a large series of out-of-hospital-cardiac arrest within the first 2 min of CPR (Beger et al., 2019) which corresponds to a first peak in the incidence of thoracic injuries (Baubin et al., 1999).

Therefore, changes due to injuries in the biomechanical characteristics of the thorax as CPR maneuvers progress could affect the elastic properties of the thorax. These changes have been associated as well with the loss of damping forces, decreasing the efficacy of CPR (Jalali et al., 2017).

### Effects of Serious Thoracic Injuries on Compliance of the Chest Wall and the Lung During Cardiopulmonary Resuscitation

In a cadaveric human-based model using Thiel-embalmed cadavers, the respiratory system compliance was obtained during CPR and compared with data from out-of-hospital cardiac arrest patients under mechanical ventilation (Charbonney et al., 2018). In this model, the average compliance of the respiratory system was 42 ± 12 ml/cmH_2_O and the average compliance of chest wall was 110 ± 45 ml/cmH_2_O, which was comparable with measures taken from out-of-hospital cardiac arrest patients.

Unilateral rib fractures, even if not associated with parenchymal injuries, have been associated with a significant decrease in respiratory system compliance in critically ill patients receiving mechanical ventilation (Cinnella et al., 2001). On the other hand, the static compliance of the respiratory system decreases after the establishment of cardiorespiratory arrest and is accentuated with the duration of CPR maneuvers. This seems to be associated with the presence of alveolar collapse and atelectasis, which reduce lung volume (Davis et al., 1995).

**Figure 1:**
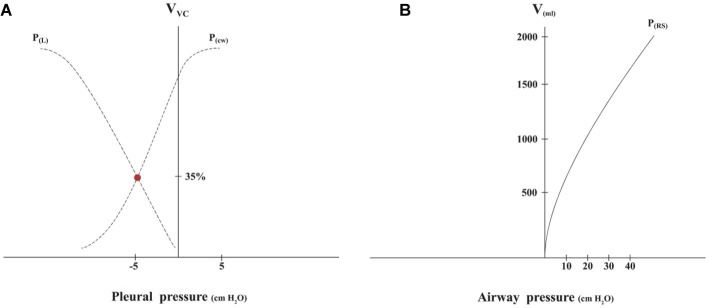
**(A)** The Campbell diagram is obtained sequentially measuring pressure-volume points without airflow. The curves that are obtained in a Cartesian coordinate axis are the lung pressure-volume curve [P(L)] and chest wall pressure-volume curve [P(cw)]. The x-axis represents the pleural pressure. When this is equal to zero, it is the same as the atmospheric pressure. The y-axis represents volume (V) expressed in % of vital capacity (VC). The point where the curves cross corresponds to the functional residual capacity (FRC); at this point, where the chest wall tends to turn outward, while the lungs tend to turn inward, is the end of expiration. **(B)** The pressure-volume curve of the respiratory system (RS) is obtained measuring several measures of the plateau pressures with different volumes inflated, which are increased in 250-ml increments up to 2000 ml, obtaining static compliance as previously described (Davis et al., 1995).

A CD is a theoretical framework that aids in understanding the relationships between pressure, volume, and compliance of the most important elements of the respiratory system (Figure 1A).

Due to the difficulty of measuring pleural pressure and volumes of the chest wall and the lungs separately, in clinical practice, the pressure-volume curve of the respiratory system, which includes the lungs and chest wall, is used to estimate compliance (Figure 1B). The airway pressure (represented by the x-axis) has a good correlation with pleural pressure and right atrial pressure during CPR when airflow is zero (Kwon et al., 2015).

**Figure 2:**
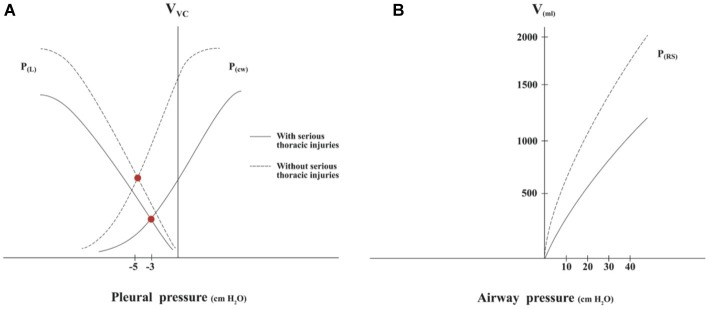
**(A)** Representation of pressure-volume curves of the lung and chest wall in the thorax with serious thoracic injuries. Compliance decrease of the chest wall and lung implies a new slope of both pressure-volume curves, which leads to a fall in functional residual capacity and a decrease in negative pleural pressure in the thoracic decompression CPR phase. **(B)** Representation of pressure-volume curves of the respiratory system with and without serious thoracic injuries. The average compliance depicted in the thorax without serious injuries is 51 ml/cmH_2_O and with serious injuries 24 ml/cmH_2_O. Based on data from Davis et al., 1995 and Cinnella et al., 2001.

When representing the fall in compliance in both pressure-volume curves of the CD, a new balance point is established with a new, lower functional residual capacity (FRC) and a higher pleural pressure (Figure 2A). This new volume and pressure operation point of the CD corresponds to the thorax in the decompression phase when there is no ventilation during CPR. The reduction in the FRC was described previously in a thorax with multiple fractures (Lewis et al., 1975).

In the pressure-volume curve of the respiratory system, a shift to the right can be observed as a result of the reduced compliance due to thoracic injuries (Figure 2B).

### Hemodynamic Effects of a Decrease in Chest Wall and Lung Compliance

The effectiveness of chest compressions is determined by the restoration of the venous return gradient. This flow depends on the gradient between the mean systemic filling pressure of the venous system and the right atrial pressure (Chalkias et al., 2019). The “suction” effect that favors venous return is generated by the negative pressure created when the chest wall recovers its position during decompression; this is one of the main elements of the “thoracic pump” theory. This phenomenon is affected by the loss of respiratory system compliance and the decrease in FRC, as shown in the CD.

In an animal model, the group with fewer rib fractures had better thoracic recoil, and on average a 5 mm greater anteroposterior diameter based on measure before and after compressions and significantly better coronary perfusion (Liao et al., 2010). In another study, chest molding and increased static chest deformity were associated with inadequate thoracic recoil and worse hemodynamic outcomes (Dean et al., 1990). Moreover, incomplete thoracic re-expansion occurred frequently (12–44% of the time) when the rescuer leaned over the thorax during thoracic decompression. This is associated with a significant increase in intrathoracic pressure accompanied by a significant decrease in cerebral perfusion and coronary pressures (Niles et al., 2011). Rib fractures, especially if they are bilateral (as in many cases of STIs), decrease thoracic elasticity (Liao et al., 2010), which could worsen the effect of leaning during CPR.

Finally, a reduced lung compliance due to STIs increases the transpulmonary pressure, which is needed during each external ventilation in CPR, and therefore has a hemodynamic impact on the right ventricular afterload. Moreover, this adverse effect on the right ventricular afterload could be worsened in the presence of the hyperventilation commonly observed during CPR. The increase in ventilation frequency from 12 to 30 pm in an animal model of cardiac arrest multiplied the mean intrathoracic pressure by 2.7 and decreased coronary perfusion pressure by 33% (Aufderheide and Lurie, 2004).

### Effects of Serious Thoracic Injuries on the Cardiac Pump

The theory of the cardiac pump is based on the direct effect of compression of the sternum over the left ventricle. When the ventricle deforms appropriately between the sternum and the anterior faces of the vertebral bodies, this effect produces an anterograde flow able to generate and sustain cardiac output during CPR (Georgiou et al., 2014).

It has been found that the anteroposterior distance covered by the sternum after 5 min of CPR with an active compression and decompression device increases significantly (Segal et al., 2017). This finding has been associated with a decrease in thoracic stiffness produced by rib fractures (Oh, 2017). The increase in the displacement involves an increase in the intrathoracic pressure during compressions. Intrathoracic pressure correlates with airway pressure measured in intubated patients. In a series of patients in cardiac arrest, values of mean airway pressure of 42.5 cmH_2_O have been associated with the return of spontaneous circulation (Chalkias et al., 2017), but pressures over 70 mbar can diminish cardiac output due to the mechanical limitation of the flow (Koeken et al., 2011).

Cardiac outflow decreases dramatically when the area of maximum compression under the sternum approaches the aortic valve, as the thoracic compressions over this area can produce an obstruction of the left ventricular outflow tract (LVOT) (Hwang et al., 2009). **A** study based on consecutive CT scans of 677 patients described that the LVOT was under the lower part of the sternum in 36.64% of the cases (Papadimitriou et al., 2013). In a recent series of patients included in an extracorporeal CPR protocol, more than 50% had a closed LVOT during chest compression, and this was associated with poor clinical outcomes (Catena et al., 2019). The sternum can produce protruding edges, and multiple rib fractures can flatten the anterior thoracic wall, leading to such intrathoracic injuries as cardiac contusions (Ihnát Rudinská et al., 2016). These may also produce an increase in the LVOT obstruction underneath the sternum.

### Avoidable Injuries and Personalized Cardiopulmonary Resuscitation

Serious rib cage damage have been defined as a sternal fracture and/or > six unilateral rib fractures and/or > four rib fractures if one of them is bilateral (Koster et al., 2017). The definition of thoracic injuries with hemodynamic adverse effects and their prevalence are still an unresolved issue.

Studies of avoidable CPR-associated thoracic injuries have been scarce in the literature, and this could be a promising new field of investigation. It has been stated that up to 20% of thoracic fractures could be avoided. Cranial displacement of the hands on the thorax can produce upper rib fractures (Krischer et al., 1987), and these could be avoided with the use of a patch to facilitate the location of the compression point. The caudal sliding of the mechanical compressor LUCAS during patient transport was associated with an increase in visceral lesions (Englund and Kongstad, 2006); therefore, a cervical strap was designed to prevent it.

In the last decade, resuscitation guidelines have attempted to establish the best balance between the benefit of increased depth of chest compressions and the risk of harm to the patient (Møller Nielsen and Rasmussen, 2013). The compression depth of 50–60 mm, recommended in the latest guidelines, has been associated with an increase in injuries (Young et al., 2011). On the other hand, the maximum survival is associated with compressions between 40.3 and 55.3 mm deep (Stiell et al., 2014). The greater vulnerability of women to STIs brings the upper limit of the most appropriate depth into question, especially in older women who have a higher prevalence of degenerative bone diseases (Champigneulle et al., 2018). A faster chest compression release has been associated with improved outcomes (Kovacs et al., 2015). There is a significant decrease in the chest compression release velocity among the older population and women during the first 10 min of CPR (Beger et al., 2019). It must be borne in mind that the anteroposterior diameter of the chest is significantly lower in women, so there is less benefit of deeper CCs (Beesems et al., 2015).

The risk of fractures depends not only on the properties of the biological materials but also on the geometry of the rib cage (Wang et al., 2005). With increasing age or obesity (Shi et al., 2014), a horizontalization of the ribs is produced, increasing the vulnerability to sustaining injuries. A tendency for patients with a thoracic perimeter greater than 101 cm to have a higher incidence of STIs has recently been described in a series of non-survivors following CPR (Azeli et al., 2019).

Performing CPR on obese patients is a real challenge for resuscitation teams. In obese patients, the heart is displaced horizontally due to the abdominal obesity (Alpert et al., 2000), so the optimal compression point could be displaced to the left. The first favorable results have already been published on the effectiveness (Anderson et al., 2016) and safety (Azeli et al., 2017) of a compression point displaced to the left and caudally, opening a promising field of investigation.

Studies that include the assessment of CPR injuries, the hemodynamic monitoring variables, and the chest mechanics during CPR, such as intrathoracic pressure or respiratory system compliance, might be helpful in the better understanding of the hemodynamic effects of injuries during CPR and moving toward personalized CPR.

## Conclusion

STIs secondary to thoracic compressions produce a decrease in the compliance of the chest wall and the lung. Representation of these effects in the Campbell diagram shows a decrease in FRC and a decrease of the negative pleural pressure in the decompression phase. The adverse hemodynamic effects of STIs affect both the thoracic pump and the cardiac pump theories. A better knowledge of the risk factors contributing to thoracic injuries due to CPR is needed, especially for those injuries that can be avoided.

## Author Contributions

YA planned the review, wrote the first draft, and was responsible for the revisions. YA and JL performed the review. YA, JL, MM, and AB discussed and commented on the draft versions, read and commented on the final version of the manuscript, agreed to its submission for publication, and agreed to be accountable for all aspects of the work.

### Conflict of Interest

The authors declare that the research was conducted in the absence of any commercial or financial relationships that could be construed as a potential conflict of interest.
